# Association of Diet With Essential Tremor: A Narrative Review

**DOI:** 10.7759/cureus.29168

**Published:** 2022-09-14

**Authors:** Anaiska Ray, Dalia A Biswas

**Affiliations:** 1 Medicine, Jawaharlal Nehru Medical College, Datta Meghe Institute of Medical Sciences, Wardha, IND; 2 Physiology, Jawaharlal Nehru Medical College, Datta Meghe Institute of Medical Sciences, Wardha, IND

**Keywords:** essential tremor management, onset of essential tremor, progression of essential tremor, hand tremor, diet and essential tremor, essential tremor

## Abstract

Essential tremor is a neurological disorder categorized by the rhythmic shaking of the upper limbs, lower limbs, neck, or head. The etiology of essential tremor is believed to be genetic variations, environmental factors, lifestyle, etc. Poor lifestyle and diet are important factors contributing to the onset of various disorders. Environment and lifestyle play a significant part in the dietary habits of an individual. Some diet components may probably be associated with the etiopathogenesis or progression of the essential tremor. Dietary habits may be a key influence on the commencement of tremors in healthy individuals. Typically, the diet of essential tremor patients is not supervised. It may also intensify the tremors in essential tremor patients. Association of the diet with the essential tremor can shed light on the root of tremor aggravating aspect and aid in diet modification in essential tremor patients. The aim of the review is to establish a relation between the diet with etiopathogenesis and the progression of essential tremor. The review includes studies providing information about essential tremor and correlating essential tremor with diet, lifestyle, environment, and genetic factors. Studies that did not provide a link to the association of essential tremor were excluded. The interpretation of the research indicated that genetic variations might be triggered due to enzymatic changes triggered by dietary patterns. Dietary components showed ambiguous, weak, strong, or no association. Essential tremor may be influenced by diet. Further research must be carried out on essential tremor patients in the nutritional domain. Physicians may monitor the diet of the essential tremor patients and record the progress of the disorder on its basis to manage the patients with essential tremor and provide better services.

## Introduction and background

Essential tremor is a clinical syndrome with pathological and genetic conditions triggering tremors in the limbs, head, neck, and voice [1]. It is the most common form of cerebellar degeneration [2]. It may also be termed a neurological disease [3]. Essential tremor is a progressive disorder that may be characterized by cerebellar-thalamic circuits and causes tremors on initiating voluntary activities [4]. The severity of essential tremor, anxiety, and depression results in a lower quality of life [5]. The prevalence of essential tremor in the general population was 0.32% in 2020 [6]. Food habits affect several functions of the body. Similarly, diet could be associated with the severity and progression of essential tremor. Alcohol consumption improves the symptoms of essential tremor while it may also pose a risk for essential tremor on excessive consumption [7]. Baseline heavy cigarette smoking can be associated with the incidence of essential tremor indicating an association with nicotine [8]. Meat cooking and consumption can be correlated to blood harmane levels in essential tremor patients [9]. GABAergic (Gamma amino-butyric acid) diet may show therapeutic effects on essential tremor and a Mediterranean diet might reduce the odds of essential tremor in patients [10-11]. Intake of antioxidants in low amounts before the onset of essential tremor might indicate a correlation [12]. Research needs to be done to find other dietary associations with essential tremor for a comprehensive analysis of the diet of essential tremor patients. The onset of essential tremor may also be due to environmental factors like pesticides, frosted glass, etc. [13]. The prevalence of essential tremor can be seen in older patients and younger patients with a family history [14]. The disorder leads to an inability to perform day-to-day activities like holding objects. Management of essential tremor using pharmacotherapy is slow and ineffective, while surgical methods of treatment are quite expensive and not readily available or feasible at times [15-16]. Surgical procedures are not preferred by essential tremor patients due to the risks involved in the modality. Thus, it is necessary to control essential tremor despite the therapeutic ways. The hypothesis stating that the diet of an individual may be responsible for increasing or reducing the risk of incidence or onset of essential tremor may be significant.

The aim of the narrative review is to appraise the association of diet with the progression of essential tremor in patients. The purpose of the study is to shed light on the correlation of food habits with the mutations in cells or degeneration of some neural tissues that may be the reason for the onset of the essential tremor. On the analysis of the relevance of the association of diet with essential tremor, the food habits of essential tremor patients may be improved. Suggestions for diet modification during the course of treatment of essential tremor patients can be proposed.

Materials and methods

Many research articles and clinical trials provide information on the essential tremor, its prevalence, and its etiology. In this narrative review, we have discussed the work till-date and suggest further studies that are required. For the purpose of the review to establish a correlation between the diet of the patient and the progression or onset of the disorder, few studies were selected for the review. On doing a PubMed search on 25 July 2022, 129 articles were found for the keywords essential tremor, nutrition, and diet in the abstract and title, out of which only 36 were selected for the review. On Google Scholar, 58 out of 48,300 articles on the related topic provided relevant information. A total of 46 studies gave relevant information for the review. The selected studies were thoroughly read and analyzed to identify a link between the progression of the severity of the disorder and the food habits or nutritional intake of essential tremor patients. The MESH terms used were essential tremor, etiopathogenesis, and diet. The keywords are the onset, severity, progression of essential tremor due to diet, and genetic factors affecting essential tremor.

**Figure 1:**
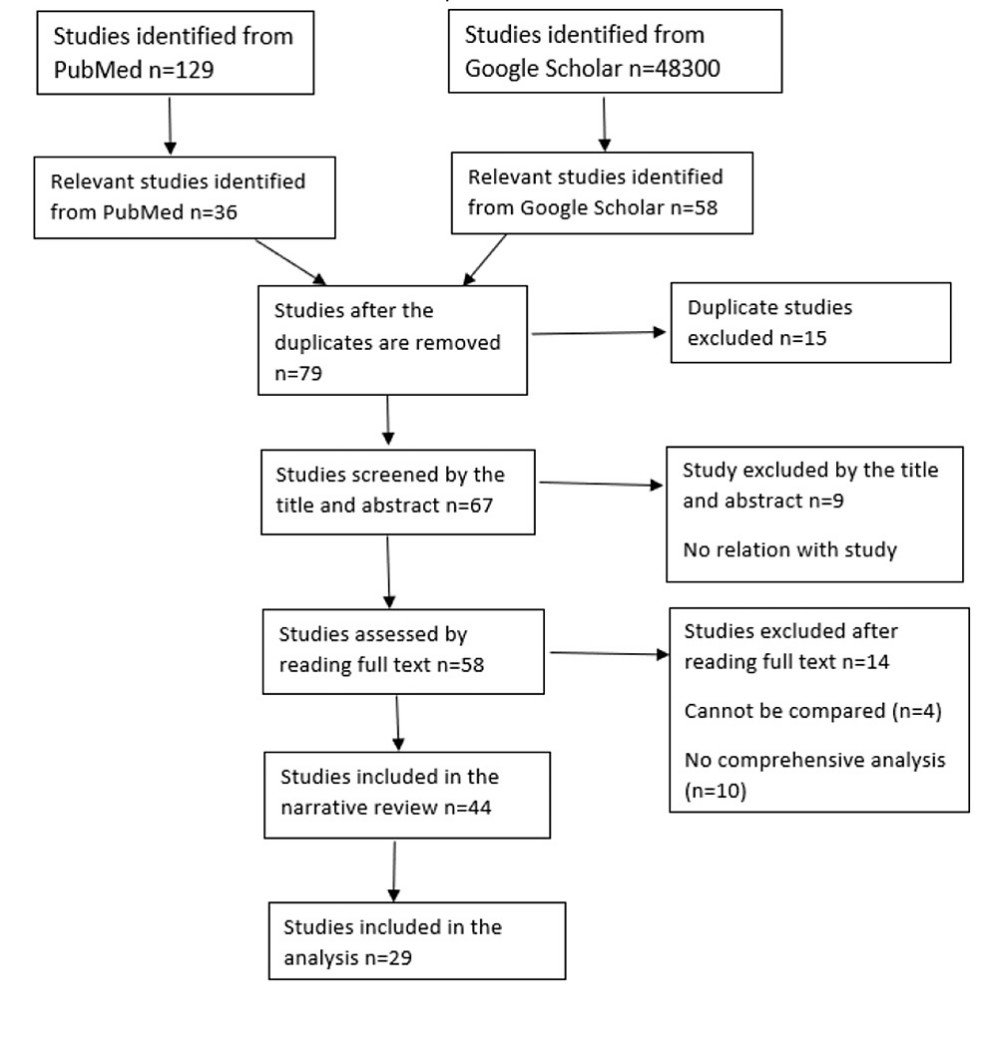
Flowchart explaining the method of the literature review.

The inclusion criteria of the review are the studies on the onset, epidemiology, and etiology of the essential tremor. The studies that gave the relevant details on the association of diet and habits of the essential tremor patients, studies on the environment and lifestyle of essential tremor patients, studies indicating the genetic variations being the cause of the essential tremor, and studies on the enzymatic risk factors, pharmacotherapy, and management of essential tremor are included. The exclusion criteria of the review are the studies not providing information regarding the epidemiology and etiology of the essential tremor. The studies that do not discuss the diet of the essential tremor patients, reports correlating essential tremor with Parkinson’s disease, studies that provided no relevant association of a dietary component with essential tremor, and studies about the surgical management of essential tremor are excluded (Figure 1).

The narrative review will discuss the dietary aspects of the essential tremor patients and nutritional components that might bring degenerative genetic changes responsible for essential tremor. The onset of essential tremor may be correlated with some components of the diet that might trigger tremors. The severity of essential tremor may be amplified due to the diet of a patient. There may be a need for modification in the diet plan of an essential tremor patient.

## Review

Risk factors for essential tremor related to diet

Environmental products composed of iron-manganese alloys containing lead and mercury amplified the risk of essential tremor. Pesticides such as organochlorine pesticides consumed through vegetables or contaminated can induce essential tremor [17]. Water from well, pesticides, cigarette smoking, frosted glass, smelting, and paint may be responsible for aggravating the severity of essential tremor [13]. Excessive ethanol consumption can cause toxicity in cerebellar tissues. The depression of the cerebellar function due to chronic ethanol consumption lowers the threshold for the risk of essential tremor. Ethanol, used for symptomatic relief, acts as a neurotoxin and has adverse effects on essential tremor patients [18]. There is no correlation between the severity of essential tremor and ethanol consumption, debunking the theory of self-medication in patients [19]. A high concentration of beta-carboline might be associated with essential tremor [20]. Harmane is a beta-carboline alkaloid chiefly found in cooked meat, coffee, etc. Cooked meat increases the blood harmane concentration. It might indicate that harmane could be an etiology for essential tremor [9]. Blood harmane and harmaline concentrations are mostly elevated in essential tremor patients [21]. Blood harmane acts like a neurotoxin and damages the cerebellar cortex specifically, which implies an emerging link between harmane concentration and essential tremor [22]. Blood harmane levels are raised on the consumption of animal protein [23]. Animal protein intake may provide a link to the etiology of the onset of essential tremor. Individuals may be exposed to the inorganic or organic form of lead which might be damaging [15]. Blood lead concentration is associated with the incidence of the neurological disorder essential tremor, while studies need to assess the efficacy of blood lead concentration in the pre-disease state to determine the increased risk of developing essential tremor [24-25]. The delta-aminolaevulinic acid dehydratase gene codes for the crucial enzyme responsible for lead metabolism, and interaction among the polymorphic gene and blood lead might increase the chances of essential tremor [26]. Blood lead levels are elevated by lower consumption of dietary components vitamin C, calcium, and iron, while bone lead concentration is increased by inadequate intake of vitamin D [27]. Therefore, there might be an indirect correlation between the diet and essential tremor for the elevated levels of lead concentration (Table 1).

**Table 1:** Summary of the risk factors of essential tremor related to diet.

S. No.	Title	Authors	Year of study	Type of study	Outcome
1.	Environmental epidemiology of essential tremor [17]	Louis	2008	Review	Pesticides may act as a neurotoxin responsible for preliminary prosperity of essential tremor but are not yet fully established
2.	Environmental risk factors for essential tremor [13]	Jiménez-Jiménez et al.	2007	Multivariate study	Can establish an association between exposure to frosted glass, agricultural work, cigarettes, alcohol, and the onset of essential tremor
3.	Population-based study of baseline ethanol consumption and risk of incident essential tremor [18]	Louis et al.	2009	Population-based study	Higher levels of ethanol consumption increased the risk of essential tremor
4.	Semi-quantitative data on ethanol consumption in 354 et cases and 370 controls [19]	Louis and Michalec	2014	Clinical epidemiological study	There is no correlation between ethanol consumption and essential tremor severity
5.	Elevation of blood β-carboline alkaloids in essential tremor [20]	Louis et al.	2002	Case control study	May establish a relationship between elevated levels of blood harmane concentration and essential tremor
6.	Dietary epidemiology of essential tremor: meat consumption and meat cooking practices [11]	Louis et al.	2008	Case control study	The possible relation of neurological disorders due to diet. Further investigations are required.
7.	Relationship between blood harmane and harmine concentrations in familial essential tremor, sporadic essential tremor and controls [23]	Louis et al.	2010	Case control study	Elevated blood harmane and harmaline concentration might be due to genetic factors in familial essential tremor
8.	Blood harmane is correlated with cerebellar metabolism in essential tremor: a pilot study [22]	Louis et al.	2007	A pilot study	The emerging link between blood harmane concentration and essential tremor
9.	Blood harmane concentrations and dietary protein consumption in essential tremor [23]	Louis et al.	2005	Case control study	Metabolic defects maybe increase blood harmane levels in essential tremor
10.	Association between essential tremor and blood lead concentration. [24]	Louis et al.	2003	Case control study	Can establish an association between blood lead concentration and essential tremor
11.	Elevated blood lead concentrations in essential tremor: a case-control study in Mersin, Turkey [25]	Dogu et al.	2007	Case control study	Association of blood lead, a neurotoxin, and essential tremor can be established
12.	Interaction between blood lead concentration and δ‐amino‐levulinic acid dehydratase gene polymorphisms increases the odds of essential tremor [26]	Louis et al.	2005	Case control study	Circulating blood lead concentrations can contribute to the incidence of essential tremor
13.	Relation of nutrition to bone lead and blood lead levels in middle-aged to elderly men [27]	Cheng et al.	1998	Normative aging study	Low vitamin D intake may increase lead accumulation in bones while low intake of vitamin C may increase lead levels in the blood

Relation of genetic risk factors to diet

The genetic risk factors involve the mutation in the genes that regulate some metabolic functions in essential tremor. A study presented an observation on the amino acid concentration fluctuation in serum exhibiting opposite changes in the concentrations of aspartate, serine, tyrosine, leucine, and isoleucine in serum independent of the concentrations in the cerebrospinal fluid of essential tremor patients. The increase in the concentration of glutamate and decline in levels of Gamma amino-butyric acid (GABA), glycine, and serine in cerebrospinal fluid may initiate a neurochemical basis of central oscillation seen in essential tremor. The metabolic disorder determined by the genetic changes might be responsible for the onset of essential tremor [28]. The pathophysiology of essential tremor involves the cortico-olivary-cerebellar-thalamic circuit. Defect in the genes and studies of single nucleotide polymorphism through genotyping concluded olivary dysfunction along with cerebellar degeneration and GABAergic degeneration [29]. Genes such as LINGO1 rs9652490 and STK32B rs10937625 may influence the incidence of essential tremor [30]. MAPT H1 haplotype is a risk factor for the incidence of essential tremor [31]. Bioactive compounds influence the expression of the gene. Further research needs to be done to understand the role of nutrigenomics in essential tremor for dieticians to give more effective dietary recommendations to essential tremor patients. Heme oxygenase 1rs2071746 and Heme oxygenase 2 rs1051308 polymorphisms can be linked to the cerebellar neurodegenerative model of the pathogenesis of essential tremor [30]. Food such as curcumin and flavonoids induce the expression of these genes and might be avoided to lessen the risk of incidence of essential tremor [32] (Table 2).

**Table 2:** Summary of the relation of genetic risk factors with diet. CSF, cerebrospinal fluid

S. No.	Title	Authors	Year of study	Type of study	Outcome
1.	Change in the concentrations of amino acids in CSF and serum of patients with essential tremor [28]	Málly et al.	1996	Observational study	Change in concentration of amino acid in cerebrospinal fluid of essential tremor results in central oscillation
2.	The etiology of essential tremor: genes vs. environment [29]	Hopfner and Helmich	2018	Review	Genetic and environmental factors may result in abnormal cortico-olivo-cerebello-thalamic activity and ultimately essential tremor
3.	Genetic risk factors for essential tremor: a review [30]	Siokas et al.	2020	Review	LINGO1 rs9652490 and STK32B rs10937625 appear to influence, to some extent, essential tremor susceptibility
4.	MAPT H1 haplotype is a risk factor for essential tremor and multiple system atrophy [31]	Vilariño-Güell et al.	2011	Case control study	MAPT H1 haplotype is a risk factor for essential tremor patients
5.	Heme oxygenase 1 and 2 common genetic variants and risk for essential tremor [32]	Ayuso et al.	2015	Observational study	A weak association between *HMOX1* rs2071746 and *HMOX2* rs1051308 polymorphisms and the risk of developing essential tremor
6.	Eat to heal: natural inducers of the heme oxygenase-1 system [33]	Correa-Costa and Otterbein	2014	Experimental study	Some dietary components upregulate the heme oxygenase-1

Epidemiology of essential tremor

The onset of essential tremor is at a young age when there is a significant family history of essential tremor. A family history of essential tremor increases the risk of the disorder at an early age [13]. The familial form of essential tremor has a narrow phenotype compared to the sporadic form and is categorized by two different chromosome locations [34]. The sporadic form of essential tremor is common in older people. The progression of the neurological disorder is rapid in the onset of essential tremor at an older age [14]. Water from the well or rural living does not associate with the onset of essential tremor, indicating no correlation of rural diet with essential tremor while it is prevalent in the white race signifying a link to the diet of the white race in the onset of essential tremor [35]. Research needs to be done in the aspect to confer the interpretation. The association of hand tremor severity with midline tremor is stronger for males than females, while severe hand tremor in females may increase the incidence of voice and head tremors [36]. A dopamine variant D3 is linked to the risk of incidence and age of onset of essential tremor [37]. 

Management of essential tremor

Dopamine receptor D3 partial agonists or antagonists inducing diet may be considered therapeutic options for patients with essential tremor [37]. Surgical treatment modalities such as magnetic resonance-guided focused ultrasound thalamotomy, deep brain stimulation, and radiosurgery improve the quality of life in essential tremor patients [38-39]. The prevalence of essential tremor can be reduced by the use of certain drugs. Pharmacotherapy of essential tremor reduces the risk of incidence. The drugs clinically relevant for the treatment of essential tremor include propranolol, primidone, and topiramate; alprazolam, clonazepam, chemo denervation with botulinum toxin, beta-adrenergic blockers, gabapentin, carbonic anhydrase inhibitors, clozapine, flunarizine, clonidine, nimodipine, pregabalin, gabapentin, and theophylline may also be beneficial [15, 40-41]. Around 50% of patients have a reduction in the severity of essential tremor with the help of propranolol and primidone [40]. Invasive surgical treatments and radiotherapy may not be effective for the long term or desired by the patient due to their risks [40, 42]. Deep brain stimulation is effective, safe, and a good option for long-term treatment. It is the standard form of surgical treatment which leads to improved quality of life by 33% [40]. It is necessary to have essential tremor in control and prevent the risk of recurrence in familial essential tremor for a better quality of life. It could be possible through a modified diet for essential tremor recovered patients.

Improvement of the condition of essential tremor through diet

The GABAergic diet includes food rich in glutamic acid such as soy protein, fermented yogurt, spinach, sweet potato, oranges, citrus fruits, etc. It might be associated positively with the therapeutic effects of essential tremor by depressing the symptoms and improving the condition [10]. A Mediterranean diet, which involves ethanol consumption, smoking, meat consumption, and coffee intake, helps relieve the symptoms of essential tremor and improves the condition of the patients, that is decreasing the tremors. There has been less evidence of the Mediterranean diet aggravating the essential tremor or triggering its progression [11]. Alcohol provides symptomatic relief in tremor amplitude when it is taken in a limited amount [7]. A study reported that caffeine consumption has no correlation to the severity of tremors in patients [43]. Caffeine intake in coffee and tea may not have an association with essential tremor, but a study categorizes it as protective in action for the disorder [44-45]. It may or may not be associated with essential tremor. Studies providing evidence for the protective behavior of caffeine are necessary to establish a correlation. Nicotine might be protective in essential tremor as it protects against degenerative effects [17]. Tobacco contains nicotine which might portray the significance of cigarette smoking in essential tremor patients. There have been contradictory reports on cigarette smoking as an aid to the progression of the severity of tremors and baseline heavy smoking showing a lower risk of incidence of essential tremor [8, 46]. However, it is not an advisable dietary component due to the risk of addiction and the adverse effects of nicotine on human health. The effects of all the dietary components can be summarized as follows (Table 3). 

**Table 3:** Effects of diet on essential tremor. GABA, gamma amino-butyric acid

Diet components	Triggering effects	Relieving effects	Association
Caffeine (coffee, tea, etc.) [44-45]	Nil	May be protective	Ambiguous
Alcohol [7]	On excessive consumption	Intake in limited amounts	Ethanol/alcohol affects symptoms
Nicotine (cigarette smoking) [8]	Not known	Not known	Lowers the risk of incidence
Meat consumption/blood harmane [9]	Might be there	Not known	Might be responsible for the onset
Mediterranean diet [11]	Does not trigger	Improves condition	Intake improves condition
MAPT H1 haplotype triggering diet	Effects seen	Nil	Ambiguous
GABAergic diet	Nil	Improves condition	Strong Association
Antioxidant	No evidence	Might be protective	May be correlated
Heme oxygenase 1 and 2 triggering diet [32]	Fewer effects	Not known	Weak association

Limitations of the review

The limitation of this narrative review is the insufficient information on the association of diet with essential tremor. There are very few clinically acclaimed studies on the specific dietary component affecting essential tremor. The limitation of the present studies on essential tremor and diet is that there is no concrete basis for a correlation between food habits with tremor severity. The controls in all the studies on environmental factors and lifestyle influencing essential tremor do not deal with a particular component exclusively resulting in adulteration of the data due to other factors. There is no investigation of the gender-specific or age-specific carbohydrate, protein, and lipid levels in the essential tremor patients to establish a link. Some other articles were not specific to essential tremor and discussed neurological disorders in general. Those studies could not be included in this narrative review. The data of some studies are ambiguous to provide a link between essential tremor and the dietary component. Most of the studies try to prove a hypothesis with a single conclusion which may not be notable for correlation with the diet of essential tremor patients. There is no sex differentiation or gender-specific discussion in our narrative review, however, it does not disturb the conclusion of the study.

## Conclusions

The review was on the association of essential tremor with diet. We related studies to establish or refuse the association of dietary components with the risk of incidence, onset, and progression of essential tremor. Herewith, there is no concrete evidence of the progress of essential tremor based on familial history while it worsens gradually in older patients who acquired the disorder. The management of essential tremor might involve a change in lifestyle and diet of the patient to avert the advancement of the disorder. The association of the Mediterranean diet, adequate ethanol consumption, cigarette smoking, GABAergic diet, and caffeine consumption showed somewhat positive effects, that is, decreased symptoms of essential tremor. A diet that might influence enzymatic changes resulting in mutations, excessive ethanol consumption, increase in blood harmane levels due to meat consumption showed triggering effects on the onset and progression of essential tremor. Similarly, low amounts of antioxidants in diet before the onset of essential tremor may show an association with tremor progression. Few diet components showed a weak or ambiguous association with the risk of incidence of essential tremor. And some dietary components showed a strong relationship with the increase or decrease in the onset or the severity of the essential tremor. Further research needs to be done on the association of nutritional components and vitamin deficiency with the essential tremor. Physicians might monitor the diet of essential tremor patients for some time to assess the effects of certain diets on the patient to improve the condition. Research needs to be done on the effects of specific nutritional components on essential tremor with age-specific and gender-specific studies, including the psychological factors. The diet of pre-diseased individuals with the risk of essential tremor may be analyzed to find a correlation between the onset of the disorder and food habits. The diet of the essential tremor patients may be analyzed to identify the dietary component that might trigger the tremor severity. Essential tremor patients might have to acclimatize with a few lifestyle modifications to observe improvement in their conditions while following a modified diet if an association of diet with tremor severity is established. Additional investigations are required to establish a strong association of diet with essential tremor.
